# Study of the Plant COPII Vesicle Coat Subunits by Functional Complementation of Yeast *Saccharomyces cerevisiae* Mutants

**DOI:** 10.1371/journal.pone.0090072

**Published:** 2014-02-25

**Authors:** Johan-Owen De Craene, Fanny Courte, Bruno Rinaldi, Chantal Fitterer, Mari Carmen Herranz, Corinne Schmitt-Keichinger, Christophe Ritzenthaler, Sylvie Friant

**Affiliations:** 1 Department of Molecular and Cellular Genetics, UMR7156, Université de Strasbourg and CNRS, Strasbourg, France; 2 Institut de Biologie Moléculaire des Plantes (IBMP), UPR2357 CNRS, Université de Strasbourg, Strasbourg, France; Institute of Molecular and Cell Biology, Biopolis, United States of America

## Abstract

The formation and budding of endoplasmic reticulum ER-derived vesicles depends on the COPII coat protein complex that was first identified in yeast *Saccharomyces cerevisiae*. The ER-associated Sec12 and the Sar1 GTPase initiate the COPII coat formation by recruiting the Sec23–Sec24 heterodimer following the subsequent recruitment of the Sec13–Sec31 heterotetramer. In yeast, there is usually one gene encoding each COPII protein and these proteins are essential for yeast viability, whereas the plant genome encodes multiple isoforms of all COPII subunits. Here, we used a systematic yeast complementation assay to assess the functionality of *Arabidopsis thaliana* COPII proteins. In this study, the different plant COPII subunits were expressed in their corresponding temperature-sensitive yeast mutant strain to complement their thermosensitivity and secretion phenotypes. Secretion was assessed using two different yeast cargos: the soluble α-factor pheromone and the membranous v-SNARE (vesicle-soluble NSF (N-ethylmaleimide-sensitive factor) attachment protein receptor) Snc1 involved in the fusion of the secretory vesicles with the plasma membrane. This complementation study allowed the identification of functional *A. thaliana* COPII proteins for the Sec12, Sar1, Sec24 and Sec13 subunits that could represent an active COPII complex in plant cells. Moreover, we found that AtSec12 and AtSec23 were co-immunoprecipitated with AtSar1 in total cell extract of 15 day-old seedlings of *A. thaliana*. This demonstrates that AtSar1, AtSec12 and AtSec23 can form a protein complex that might represent an active COPII complex in plant cells.

## Introduction

The formation and loading of endoplasmic reticulum ER-derived vesicles is a highly conserved function in eukaryotic cells and requires a vesicular coat termed the coat protein complex II (COPII) that was first identified in yeast *Saccharomyces cerevisiae* [1], [2]. The COPII coat is constituted of five cytoplasmic proteins Sar1, Sec13, Sec23, Sec24 and Sec31 and one ER resident transmembrane protein Sec12. Together they participate to the stepwise COPII vesicle assembly process [3]. Vesicle formation is initiated by the recruitment of the Sar1 GTPase to the ER membrane and its activation by the guanine exchange factor (GEF) Sec12. Activated Sar1 interacts with the Sec23–Sec24 heterodimeric complex, which recruits the cargos and forms a subregion of the ER membrane enriched in COPII proteins. This results in the recruitment of the Sec13–Sec31 heterotetramer by the Sec23–Sec24 complex and induces ER membrane bending [4]. After vesicle formation and prior to vesicle fusion with the Golgi, the vesicle uncoats after GTP hydrolysis by Sar1 stimulated by the GTPase activating protein domain of Sec23 [5].

In yeast there is only one protein of each family to fulfill these functions, except for Sec24 which has two additional Sec24-like proteins involved in the active recognition of the different sorting signals found on cargo proteins [6], [7] (Fig. S1). In metazoan, each COPII subunit has two isoforms, except Sec13, which has none, and Sec24, which has four termed Sec24A, B, C and D [8]. In plants, there are even more predicted isoforms than in metazoan (Fig. S1), rendering their systematic analysis very complicated and raising also questions about possible overlapping functions, tissue specificity of certain complexes and the rules of subunit association [9], [10].

Here, we performed a systematic complementation assay to functionally analyze *Arabidopsis thaliana* COPII subunits in the yeast *Saccharomyces cerevisiae*. This study was undertaken in this system because complementation of the temperature-sensitive phenotype of yeast mutants has been successfully used to analyze *in vivo* COPII subunits from different eukaryotic organisms including plants [11], [12], [13], [14], [15]. Furthermore, from an evolutionary point of view, all COPII subunits stem from bacterial proteins bearing a WD40 domain and share a common molecular architecture [16]. In this study, the different *A. thaliana* COPII subunits were expressed in their corresponding temperature-sensitive *S. cerevisiae* mutant strain, we analyzed complementation of the thermosensitive growth and of the secretion defect by using the soluble yeast α-factor pheromone and the plasma membrane SNARE (soluble NSF (N-ethylmaleimide-sensitive factor) attachment protein receptor) Snc1. This complementation study allowed the identification of functional *A. thaliana* COPII proteins for the Sec12, Sar1, Sec24 and Sec13 subunits that could form an active COPII complex in plant cells. We could also identify an association between AtSar1, AtSec12 and AtSec23 *in vivo* in plants cells by co-immunoprecipitation with an anti-AtSar1 serum.

## Results

### Functional Analysis of the AtSec12 Protein in Yeast

**Figure 1:**
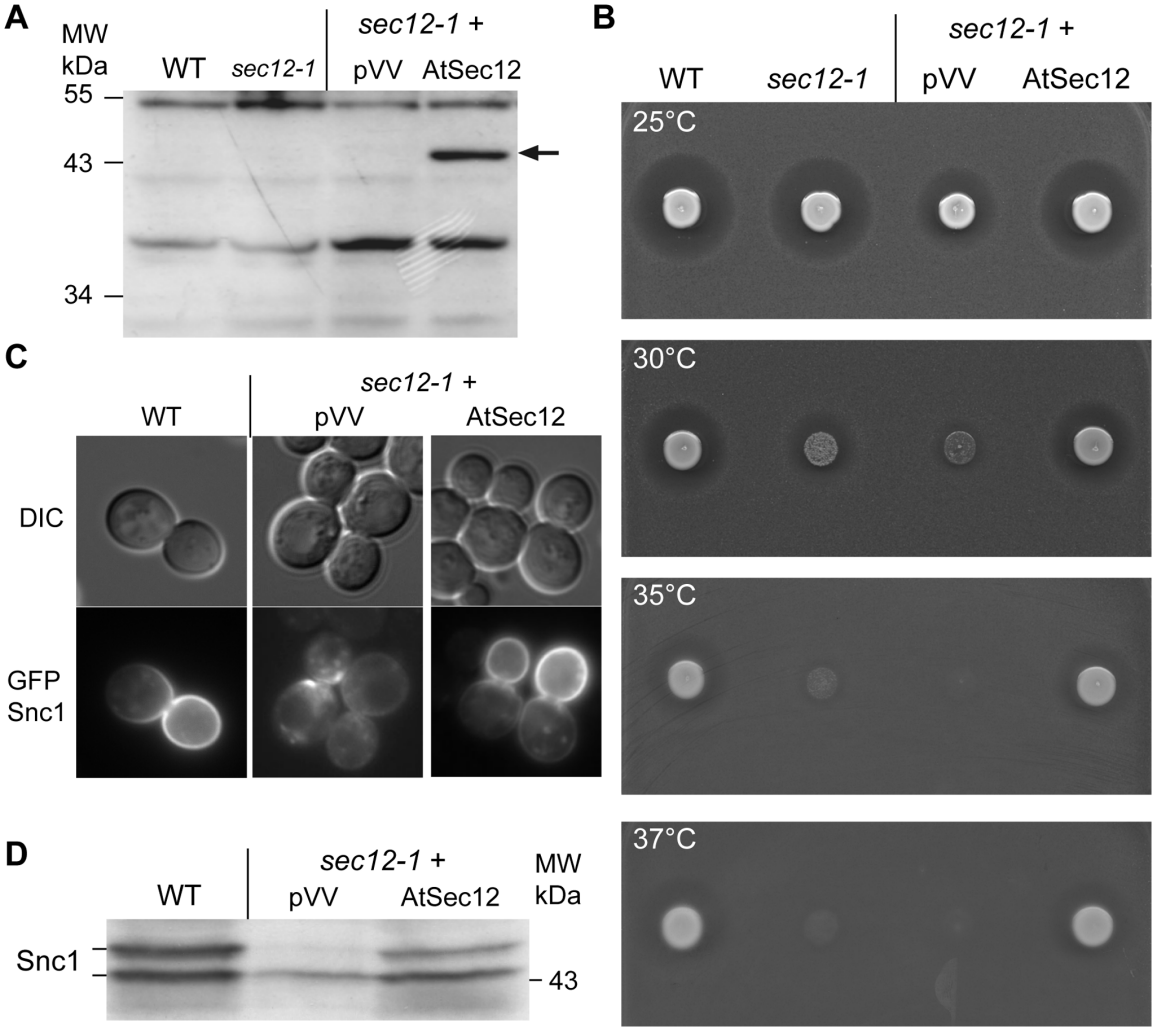
Suppression of the yeast *sec12-1* by the plant AtSec12 homologue. A) Total protein extracts of yeast wild-type (WT) and *sec12-1* mutant cells untransformed or transformed with the empty vector (pVV) or expressing plant *AtSEC12* were resolved by SDS-PAGE and immunoblotted with anti-AtSec12 antibodies. B) Yeast cell cultures grown to mid-exponential phase at 25°C were spotted on YPD medium containing *MATa bar1* mutant cells to determine α-factor secretion at permissive (25°C) and various restrictive temperatures (30°C, 35°C and 37°C). C) Localization of a COPII cargo the SNARE GFP-Snc1 in the *sec12-1* mutant transformed with either pVV (empty) or AtSec12 plasmids was determined by epifluorescence observation (with GFP and Differential interference contrast (DIC) filters) on cell cultures grown to mid-exponential phase at 25°C. D) Total protein extracts from wild-type (WT) cells or *sec12-1* mutant cells transformed with pVV or AtSEC12 plasmid and with GFP-Snc1 vector and grown at 25°C were resolved by SDS-PAGE and immunoblotted with anti-GFP antibodies.

We started our functional analysis of *A. thaliana* COPII proteins in yeast with the analysis of the Sec12 ER transmembrane protein homolog: At2g01470 (AtSec12, 393 aa, also termed STL2P). Indeed, this AtSec12 protein was shown to complement the *sec12* yeast mutant [11] and AtSec12 is involved in COPII dependent ER to Golgi trafficking in plants [17]. Our phylogenetic analysis (Fig. S1) revealed two proteins related to AtSec12, the AtSec12-like protein (AtSec12L, At5g50550, 383 aa) and the AtSec12-like protein 1 (AtSec12L1, At3g52190, 398 aa, also termed PHF1); these two Sec12-like proteins were not included in our analysis. We transformed the temperature-sensitive *sec12*-*1* mutant [1], with the plasmid encoding AtSec12 under the control of the bacterial tetracycline-repressible *tetO* promoter (pVV208+AtSec12). We first performed an immunoblot assay to ascertain that the AtSec12 protein was produced in yeast cells using antibodies specifically raised against AtSec12 [18]. In the *sec12*-*1* mutant transformed with the recombinant plasmid a band corresponding to the molecular weight of AtSec12 (43 kDa) was observed (Fig. 1A), no corresponding signal was detected in untransformed yeasts or in yeasts transformed with the empty plasmid (Fig. 1A). As previously observed [11], the AtSec12 protein efficiently suppressed the temperature-sensitive growth phenotype displayed by yeast *sec12-1* mutant cells and this until 37°C (Fig. S1 and 1B).

To decipher whether the plant protein also restored secretion, we analyzed the secretory pathway in *sec12-1* yeast cells expressing AtSec12. We tested two different cargos, the soluble α-factor pheromone secreted by haploid *MATa* yeast cells, and the transmembrane receptor Snc1, a v-SNARE protein that is required for fusion between Golgi-derived secretory vesicles and the plasma membrane [19]. Indeed, soluble and membrane protein cargos do not entirely require the same COPII sorting machinery, since the soluble α-factor protein depends on the Erv29 transmembrane cargo receptor for its efficient packaging into COPII vesicles [20], whereas SNARE membrane proteins contain specific sorting signals into their sequence [21], [22]. α-factor secretion was tested by the halo test, which relies on the growth inhibition of *bar1* mutant cells induced by α-factor (Fig. 1B). The *BAR1* gene encodes an aspartyl protease secreted into the periplasmic space of *MATa* yeast cells where it cleaves and inactivates the α-factor, hence allowing cells to recover from α-factor-induced cell cycle arrest [23], [24]. In our test, the α-factor produced by *MATα* yeast cells inhibits the growth of the *bar1* mutant cells embedded in the solid medium unless secretion of the α-factor is deficient. Efficient secretion of the α-factor is assessed by the formation of a halo around the growing *MATα* yeast cells. *MATα sec12-1* cells transformed or not by the pVV208 or pVV208-AtSec12 were spotted on *bar1*-plates and incubated at permissive (25°C) or non-permissive temperatures (30°C, 35°C and 37°C). As expected, an α-factor secretion halo was observed around wild-type yeast cells at all temperatures. In contrast, the *sec12-1* mutant untransformed or bearing the empty vector pVV208 displayed a growth inhibition halo at the permissive temperature (25°C), and this halo shrank as temperature increased (Fig. 1B). Similarly to WT yeast, *sec12-1* mutant cells expressing AtSec12 displayed a strong inhibition halo at all temperatures, showing that AtSec12 complemented not only the temperature-sensitivity of the *sec12-1* mutant but also the α-factor pheromone secretion defect.

Finally, we analyzed the GFP-tagged (green fluorescent protein) v-SNARE Snc1 secretion in *sec12-1* cells transformed or not by pVV208-AtSec12 at permissive temperature. In the wild-type yeast cells, GFP-Snc1 is found at the plasma membrane and towards the bud since secretion is polarized in yeast (Fig. 1C) [25]. In *sec12-1* mutant cells, GFP-Snc1 is mainly found in intracellular structures, whereas upon AtSec12 expression, GFP-Snc1 is polarized to the bud (Fig. 1C). From the same cultures, we prepared total protein extracts and performed an immunoblot analysis to assess the phosphorylation status of GFP-Snc1 (Fig. 1D). This provides a convenient assay to monitor the secretion of GFP-Snc1. The cellular localization of GFP-Snc1 correlates with its phosphorylation status, since only the plasma membrane localized GFP-Snc1 is hyperphosphorylated [26]. The hyperphosphorylated form of GFP-Snc1 was quantified and represented 48% of total GFP-Snc1 in wild-type yeast and 40% in *sec12-1* mutant cells transformed by pVV208-AtSec12, compared to 23% in *sec12-1* cells transformed by the empty vector pVV208 (Fig. 1D). This confirmed the proper secretion of GFP-Snc1 towards the plasma membrane in *sec12-1* mutant cells expressing AtSec12 (Fig. 1D). A previous yeast complementation assay identified the AtSec12 protein as suppressing the temperature-sensitivity of the *sec12-1* mutant strain [11]. We confirmed this result by using a different expression vector. We also show that the *A. thaliana* AtSec12 efficiently complements the secretion defect of *sec12-1* yeast mutant cells and this despite being evolutionary more distant than the metazoan Sec12 homologs (Fig. S1). Our results suggest that the key functional features of Sec12 proteins are conserved throughout evolution.

### Assessment of the Yeast *sar1-2* Mutant Complementation by AtSar1 Proteins

**Figure 2:**
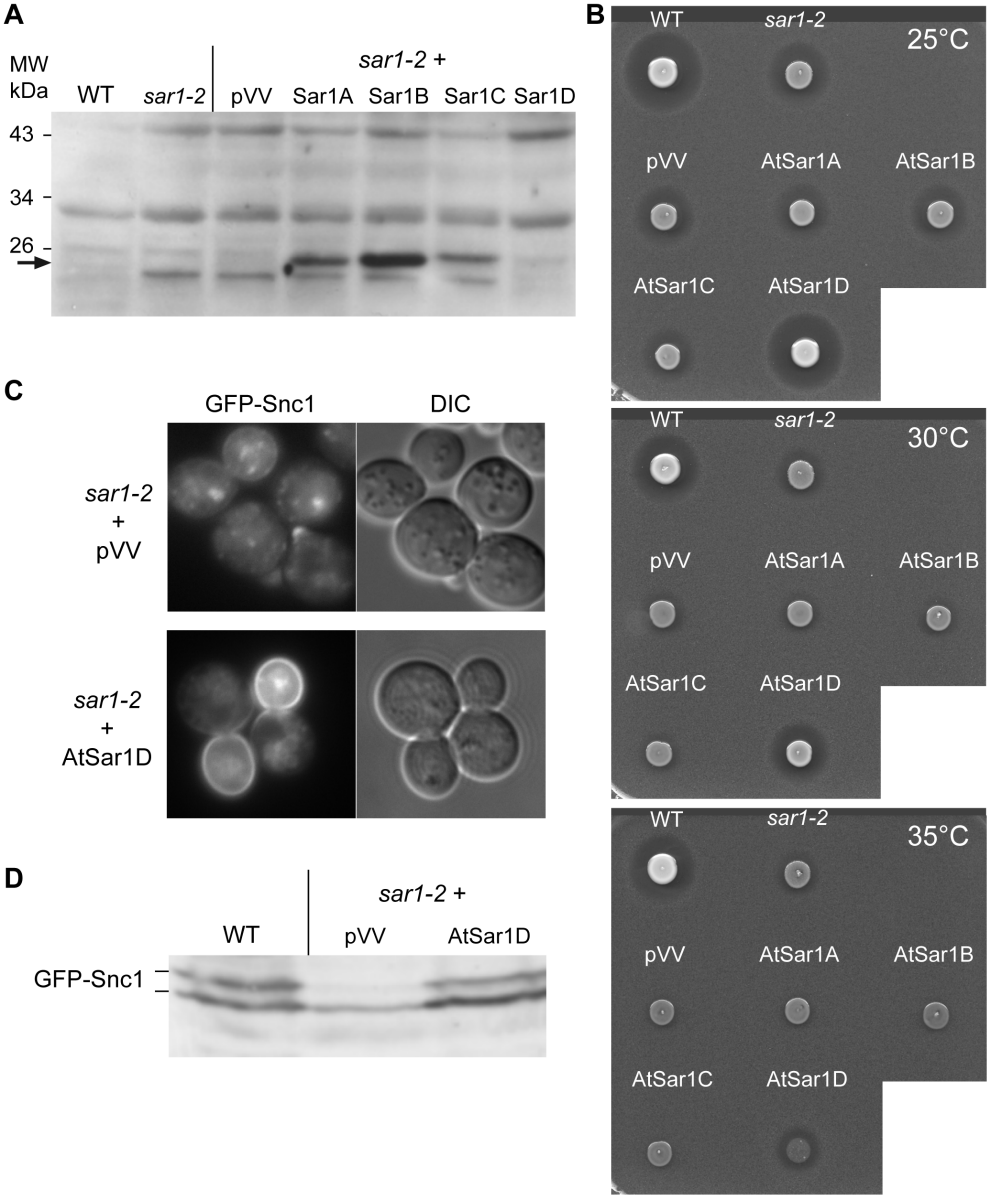
The *Arabidopsis thaliana* AtSar1D complements the secretory defect of the yeast *sar1-2* mutant cells. A) Total protein extracts of WT and *sar1-2* mutant cells untransformed or transformed with empty vector (pVV) or vectors bearing the plant *AtSAR1* isoforms A to D grown at 25°C were resolved by SDS-PAGE and immunoblotted with anti-AtSar1B antibodies. B) The same cell cultures as in A) were spotted on YPD medium containing *MATa bar1* mutant cells to analyze α-factor secretion at permissive (25°C) and various restrictive temperatures (30°C and 35°C). C) The *sar1-2* mutant cells transformed with the empty (pVV) or the AtSar1D plasmid were co-transformed with the GFP-Snc1 vector; these cells were grown at 25°C to mid-exponential phase prior their observation by fluorescence microscopy. D) Total proteins were extracted from the same cell cultures as in C) and resolved by SDS-PAGE prior immunoblotting with anti-GFP antibodies to detect the GFP-Snc1 proteins.

Having shown that our methodology worked, we next analyzed the four *A. thaliana* Sar1 paralogs At4g02080 (AtSar1A), At1g56330 (AtSar1B), At3g62560 (AtSar1C) and At1g09180 (AtSar1D). Despite showing significant sequence identity to other Sar1 proteins, AtSar1E (At1g02620) was excluded from our analysis (Fig. S1) because it is only 122 aa compared to the 193 aa of the other AtSar1 isoforms [9] and the 190 aa of the *S. cerevisiae* Sar1 protein [27]. We used the temperature-sensitive *sar1*-*2* yeast mutant for our complementation assay [28]. The *sar1-2* mutant was transformed with the pVV204 plasmids encoding AtSar1A to AtSar1D under the control of the *TetO* promoter. By immunoblot assay using antibodies raised against AtSar1B [18], we observed a 22 kDa band, the expected molecular weight of AtSar1, in *sar1-2* cells bearing AtSar1B (Fig. 2A). A similar but weaker signal at 22 kDa was also observed in yeast cells bearing AtSar1A and AtSar1C. Despite being evolutionary closest to AtSar1B (Fig. 1S), AtSar1D was barely detectable in transformed *sar1-2* cells (Fig. 2A).

We then analyzed the growth of the different transformed *sar1-2* mutant cells at different temperatures (25°C, 30°C, 35°C and 37°C) by a serial dilution drop test (Fig. S2). Among the four AtSar1 proteins, only AtSar1D ameliorated the *sar1-2* temperature-sensitive growth defect at 25°C and only up to 30°C (Fig. S2). Surprisingly in our assay, AtSar1B that was previously isolated as a multicopy suppressor of *sec12-1* [11], did not improve growth of the *sar1-2* mutant (Fig. S2). α-factor secretion was monitored by the halo inhibition assay after spotting wild-type and *sar1-2* cells transformed or not with pVV204 or pVV204+AtSar1A to AtSar1D on *bar1*-plates and incubated at 25°C (permissive temperature), 30°C and 35°C (Fig. 2B). Among the different AtSar1 proteins, only AtSar1D restored α-factor secretion at 25°C and 30°C and, despite the poor yeast growth, also clearly improved secretion of the *sar1-2* mutant at restrictive temperatures (Fig. 2B, mid and bottom panels).

Finally, we analyzed the v-SNARE GFP-Snc1 secretion in the *sar1-2* mutant transformed with pVV204 or pVV204+AtSar1D (Fig. 2C). In the *sar1-2* cells, GFP-Snc1 is mainly found intracellularly with some membrane foci, whereas in the mutant cells producing the AtSar1D, GFP-Snc1 was polarized to the bud and some intracellular patches. In parallel, we analyzed the GFP-Snc1 phosphorylation status in the different transformants. The hyperphosphorylated form of GFP-Snc1 was clearly detected in the wild-type cells and in the pVV204-AtSar1D transformed mutant, confirming the proper localization of GFP-Snc1 at the plasma membrane (Fig. 2D). The phosphorylated form of GFP-Snc1 represented 50% in the wild-type cells and 44% in the *sar1-2* mutant bearing AtSar1D, compared to 27% for the *sar1-2* mutant transformed with pVV204 (Fig. 2D). These results show that the plant AtSar1D GTPase, despite being evolutionary distant, can partially complement a defective Sar1 yeast mutant (Fig. S1).

### Analysis of AtSec23 Isoforms by Complementation of *sec23-1* Yeast Mutant

We next studied the closest Sec23 homolog At3g23660 (AtSec23A), as well as the four Sec23 paralogs At4g14160 (AtSec23B), At1g05520 (AtSec23C), At2g21630 (AtSec23D), and At5g43670 (AtSec23E). The different AtSec23 proteins were named according to their sequence similarity with AtSec23A based on the multiple alignment of complete sequences and the phylogenetic tree (Fig. S1), the closest homolog of AtSec23A bearing the B letter. Our multiple sequence alignment confirmed that the At2g27460 and At4g01810 proteins, previously described as being divergent from Sec23 and Sec24 clades [9], displayed only poor homology to Sec23 proteins. Consequently, we named them AtSec23/Sec24L1 (At2g27460) and AtSec23/Sec24L2 (At4g01810) for AtSec23/Sec24-like since they also could belong to the Sec24 family of proteins. In this study, we did not test AtSec23C (At1g05520) and the AtSec23/Sec24L2 (At4g01810) that could not be cloned. The *sec23-1* temperature-sensitive mutant strain [1], [29] was transformed with the plasmids encoding the different AtSec23 isoforms under the control of the *TetO* promoter. We did an immunoblot assay to ascertain that AtSec23B as well as the different AtSec23 proteins were present in yeast by using the polyclonal rabbit anti-AtSec23B serum [30]. Despite the poor quality of the blot, a weak band at 87 kDa was detected that corresponds to the size of the AtSec23 protein from *A. thaliana* total protein extract (Fig. S3A).

The serial dilution test showed that AtSec23A, AtSec23B and AtSec23E suppressed the thermosensitive phenotype of the *sec23-1* mutant cells up to 35°C, contrary to AtSec23D and AtSec23/24L1 (Fig. S2). Next, we tested their ability to complement the secretion defect of the *sec23-1* cells by performing a halo inhibition assay (Fig. S3B). Whereas the wild-type cells secreted α-factor at all temperatures (25°C, 30°C, 35°C and 37°C) as monitored by the clear growth inhibition halo, none of the transformed *sec23-1* mutant cells displayed an increase in *α*-factor secretion compared to the *sec23-1* mutant, and this even at 30°C (Fig. S3C). Finally, we assessed the localization at permissive temperature of the SNARE GFP-Snc1 in the *sec23-1* mutant cells transformed with the different expression vectors (Fig. S3C). In the *sec23-1* mutant carrying the empty vector (pVV), GFP-Snc1 is mainly accumulated in intracellular patches and the expression of the different AtSec23 constructs did not improve the secretion of GFP-Snc1 (Fig. S3C). The immunoblotting shows that the non-phosphorylated form of GFP-Snc1 is mainly present in the *sec23-1* cells bearing the empty vector (pVV) or transformed with the different AtSec23 constructs (Fig. S3D). These results indicate that none of the predicted Sec23 Arabidopsis isoforms can fully restore the yeast *sec23-1* secretory mutant phenotype despite a partial complementation of thermosensitivity triggered by AtSec23A, AtSec23B and AtSec23E.

### Functional Analysis of *A. thaliana* AtSec24 and AtLst1 Proteins in Yeast

The different members of the *A. thaliana* Sec24 family were considered as Sec24, Lst1 or Iss1 isoforms in previous studies [9]. Our comparative genomic analysis confirms that At3g07100 (AtSec24A) is a unique Sec24 homolog [9], [31], and shows that there are two Lst1 (also termed Sfb3) homologs At3g44340 (AtLst1A) and At4g32640 (AtLst1B). The At4g32640 (AtLst1B) protein was previously described as an Iss1 isoform, but we could not detect any clear plant homolog of the yeast Iss1 that was clustered on the same branch as Sec24 in the phylogenetic tree (Fig. S1). The yeast Iss1 (also termed Sfb2) was identified as a multicopy suppressor of *sec24* temperature-sensitive mutant and as a functional Sec24-like protein [7], [32]. Due to this discrepancy in the AtSec24 family, we expressed the different Sec24 proteins, AtSec24A, AtLst1A, AtLst1B and AtSec23/Sec24L1 (At2g27460) in the *sec24-11* and in the *lst1Δ* yeast mutant cells and tested their ability to complement the thermosensitivity, α-factor and GFP-Snc1 secretion phenotypes.

We transformed the *sec24-11* [32] and the *lst1Δ* [33] mutant strains with the plasmids encoding for AtSec24A, AtLst1A, AtLst1B and AtSec23/Sec24L1 under the control of the *TetO* promoter. We could not perform an immunoblot assay to ascertain that AtSec24 proteins are produced in yeast because there are no available antibodies raised against the *A. thaliana* Sec24 proteins.

**Figure 3:**
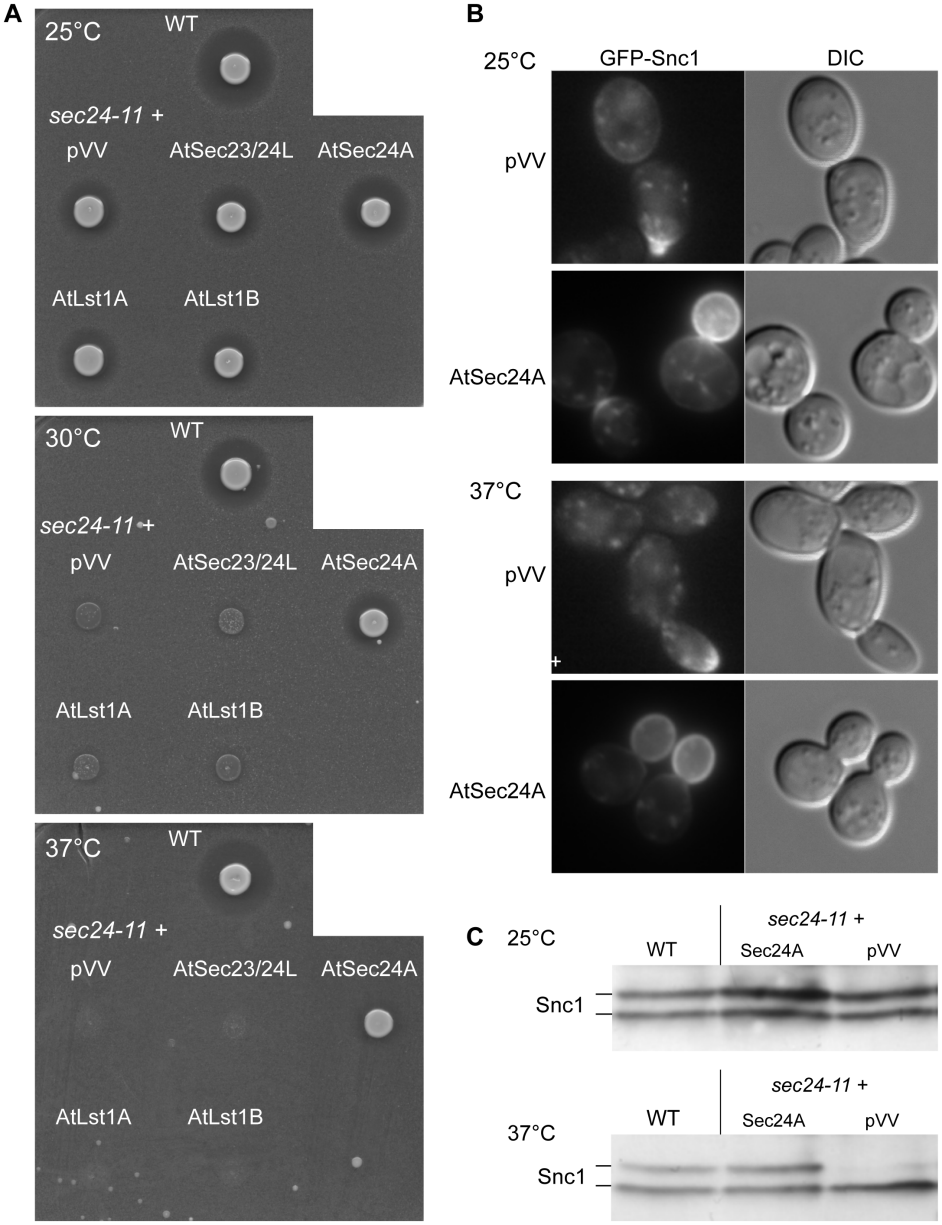
The plant AtSec24A ameliorates the phenotypes displayed by the yeast *sec24-11* mutant. A) The wild-type (WT) cells and the *sec24-11* mutant transformed with empty vector (pVV) or expressing At*SEC23/24L*, At*SEC24A* and *AtLST1A* and *B* were grown at 25°C to mid-exponential phase prior to be spotted on YPD medium containing *MATa bar1* mutant cells to determine α-factor secretion at permissive (25°C) and various restrictive temperatures (30°C and 37°C). B) The *sec24-11* mutant cells transformed with either pVV or *AtSEC24A* plasmids and expressing GFP-Snc1 were grown at 25°C to mid-exponential phase, then half of cell cultures was shifted at 25°C or 37°C for 2 h prior to their observation by fluorescence microscopy to detect the intracellular localization of the GFP-Snc1 SNARE. C) Total proteins were extracted from the same strains as in B) but after a 6 h shift at 37°C and a western-blot with anti-GFP antibodies was done to detect the state of phosphorylation of the GFP-Snc1 proteins.

We tested the complementation of the *sec24-11* thermosensitivity by a serial dilution test and observed that only AtSec24A complemented the growth of the mutant cells at 25°C, 30°C, 35°C and 37°C (Fig. S2). α-factor secretion was observed only for the *sec24-11* mutant cells expressing AtSec24A and this at 25°C and up to 37°C (Fig. 3A). Finally, we assessed SNARE GFP-Snc1 secretion at permissive temperature (25°C) and after a 2 h shift at restrictive temperature (37°C) (Fig. 3B). Indeed, contrary to the previously tested COPII temperature-sensitive mutants (*sec12-1*, *sar1-2* and *sec23-1*), the *sec24-11* cells bearing the empty vector (pVV) were not defective for Snc1-GFP secretion at 25°C, even though their α-factor secretion was affected (Fig. 3A). Therefore, we shifted the cell cultures for 2 h at 37°C prior to analysis (Fig. 3B). In the *sec24-11* mutant bearing the pVV204 empty plasmid, GFP-Snc1 was found at the plasma membrane and intracellularly at both 25°C and at 37°C. However at 37°C, the *sec24-11*+pVV204 cells displayed an elongated morphology and more intracellular accumulation of GFP-Snc1, these two phenotypes were improved in mutant cells expressing AtSec24A. To analyze the GFP-Snc1 phosphorylation status by western-blot, the cells were grown at 25°C and shifted for 6 h at restrictive temperature 37°C prior analysis (Fig. 3C). At 25°C, wild-type and *sec24-11* mutant cells displayed a high level of phosphorylated GFP-Snc1 (between 50 and 54%); after the 6 h shift at 37°C the phosphorylated form of GFP-Snc1 represented 38% of total GFP-Snc1 in the wild-type and 47% in the *sec24-11* cells bearing AtSec24A, whereas it only represented 14% in the *sec24-11* cells bearing the empty pVV204 vector (Fig. 3C). This shows that at 37°C, secretion of the Snc1 SNARE is impaired in the *sec24-11* mutant cells and that this defect is complemented by the AtSec24A protein. This demonstrates that AtSec24A is produced in yeast cells. All together, these results show that AtSec24A functionally complements the *sec24-11* mutant.

We also tested the complementation of the *lst1Δ* mutant cells by the different *A. thaliana* AtSec24 and AtLst1 paralogs (Fig. S4). None of these different paralogs AtSec24A, AtLst1A, AtLst1B and AtSec23/Sec24L1 rescued the temperature-sensitivity of the *lst1Δ* mutant cells at 37°C (Fig. S4A). Moreover, expression of these different paralogs in *lst1Δ* cells did not restore α-factor secretion at 25°C, 30°C, 35°C or 37°C (Fig. S4B) nor GFP-Snc1 secretion at 30°C (Fig. S4C and D). These results show that neither AtSec24A nor the AtLst1A/B proteins can complement the *lst1Δ* mutant.

### Functional Complementation of the *sec13-1* Yeast Mutant by the AtSec13B Protein

**Figure 4:**
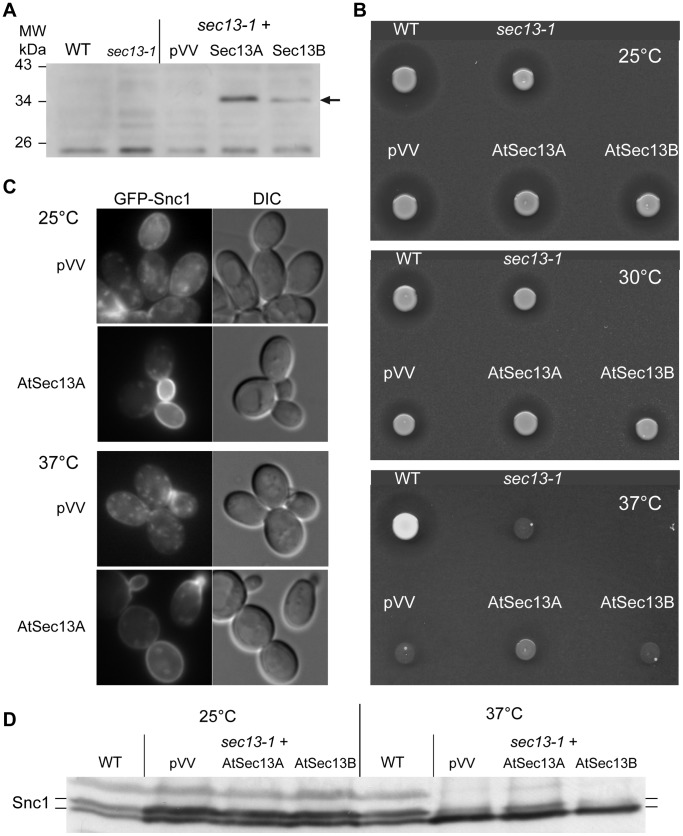
Complementation of the yeast *sec13-1* mutant by the *Arabidopsis* thaliana AtSec13A isoform. A) Yeast wild-type (WT) and *sec13-1* mutant cells untransformed or transformed with empty vector (pVV) or bearing plant AtSec13A or AtSec13B isoforms were grown at 25°C to mid-exponential phase prior lysis to extract total proteins that were subjected to western-blot analysis with anti-AtSec13A antibodies. B) The same cell cultures at in A) were spotted on YPD medium containing *MATa bar1* mutant cells to determine α-factor secretion at permissive (25°C) and different restrictive temperatures (30°C, 35°C and 37°C). C) Localization of the COPII cargo GFP-Snc1 in the *sec13-1* mutant transformed with either the empty (pVV) or AtSec13A plasmid was determined by epifluorescence on mid-exponential phase cell cultures at permissive (25°C) or after a 2 h shift at restrictive (37°C) temperature. D) Total proteins extracted from the same strains as in C) but after a 4 h shift at 37°C were resolved by SDS-PAGE and immunoblotted with anti-GFP antibodies to determine the phosphorylation status of the GFP-Snc1 proteins.

We next analyzed the two *A. thaliana* Sec13 paralogs At3g01340 (AtSec13A) and At2g30050 (AtSec13B) [9], [34]. We transformed the *sec13*-*1* temperature-sensitive yeast mutant [1] with plasmids coding for AtSec13A and -B under the control of the *TetO* promoter. The immunoblot assay using anti-AtSec13 antibodies [35] shows that the two AtSec13A and -B proteins (about 33 kDa) were produced in yeast cells (Fig. 4A).

We thus tested the ability of the two paralogs to complement the *sec13-1* temperature-sensitive growth defect by performing a serial dilution assay. We observed the efficient suppression of *sec13-1* thermosensitivity by AtSec13A up to 30°C (Fig. S2). α-factor secretion was monitored by the growth inhibition assay on the *bar1*-plates (Fig. 4B). The AtSec13A construct improved α-factor secretion of *sec13-1* mutant cells at 35°C. Finally, we assessed the secretion of the SNARE GFP-Snc1 at permissive temperature (25°C) and after a 2 h shift at restrictive temperature (37°C) (Fig. 4C). Indeed, *sec13-1* cells were not defective for α-factor or Snc1-GFP secretion at 25°C, whereas at higher temperature these cells displayed decreased secretion (Fig. 4C). After a 2 h shift at 37°C, *sec13-1* mutant cells bearing the empty pVV208 plasmid were defective for GFP-Snc1 secretion as assessed by its intracellular localization (Fig. 4C). We also monitored the phosphorylation status of GFP-Snc1 in the *sec13-1* transformants at 25°C and after a 4 h shift at 37°C (Fig. 4D). At 25°C, phosphorylated GFP-Snc1 represented between 49% and 51% of total GFP-Snc1 for the wild-type and the three different *sec13-1* transformants; at 37°C phosphorylated GFP-Snc1 represented 48% in the wild-type cells and 40% in the *sec13-1* bearing AtSec13A compared to 25% for the *sec13-1* bearing AtSec13B and 32% for *sec13-1* bearing the empty pVV208 plasmid (Fig. 4D). The *sec13-1* mutant cells expressing AtSec13A displayed clear localization of GFP-Snc1 at the plasma membrane towards the bud and an increased phosphorylation of GFP-Snc1 at 37°C (Fig. 4C and 4D). This shows that between the two AtSec13 isoforms, only AtSec13A rescues the secretion defect of the *sec13-1* yeast mutant cells.

### Analysis of AtSec31 Function

The *A. thaliana* genome encodes two Sec31paralogs At3g63460 (AtSec31A) and At1g18830 (AtSec31B) (Fig. S1) [9]. AtSec31B could not be studied because we were unable to clone it; however since AtSec31B is specifically expressed in the male organs (pollen and stamen) [9], it is less likely to form a functional complex with the other COPII subunits that present ubiquitous expression throughout the plant and are able to suppress yeast *sec-ts* mutants. The functional analysis of AtSec31A was done by transforming the *sec31-1* yeast mutant [36], with the plasmid encoding AtSec31A under the control of the *TetO* promoter. We could not perform an immunoblot assay to ascertain the production of AtSec31A in yeast cells since there are no available anti-AtSec31 antibodies.

The serial dilution test carried out at different temperatures shows that AtSec31A did not suppress the *sec31-1* mutant thermosensitivity (Fig. S2). The *sec31-1* yeast mutant cells are defective for growth and α-factor secretion at 35°C and 37°C, and these defects are not complemented by AtSec31A (Fig. S5A). Due to the weak *sec31-1* mutant phenotypes at the permissive temperature (25°C), we shifted cells at 37°C for 2 h before analyzing the GFP-Snc1 intracellular localization and phosphorylation. At 25°C, GFP-Snc1 is properly secreted in *sec31-1* untransformed cells and those bearing the pVV208 or the pVV208-AtSec31A plasmid (Fig. S5B). At 37°C, GFP-Snc1 was detected in intracellular patches in *sec31-1* yeast cells expressing AtSec31A or not. From the same cultures, total proteins were extracted and GFP-Snc1 phosphorylation was assessed showing no hyperphosphorylation of GFP-Snc1 at 37°C in the different *sec31-1* cell cultures (Fig. S5C). These results show that the *A*. *thaliana* AtSec31A paralog did not complement the *sec31-1* yeast mutant.

### Partial COPII Complex Purification in *A. thaliana* Plants

**Figure 5:**
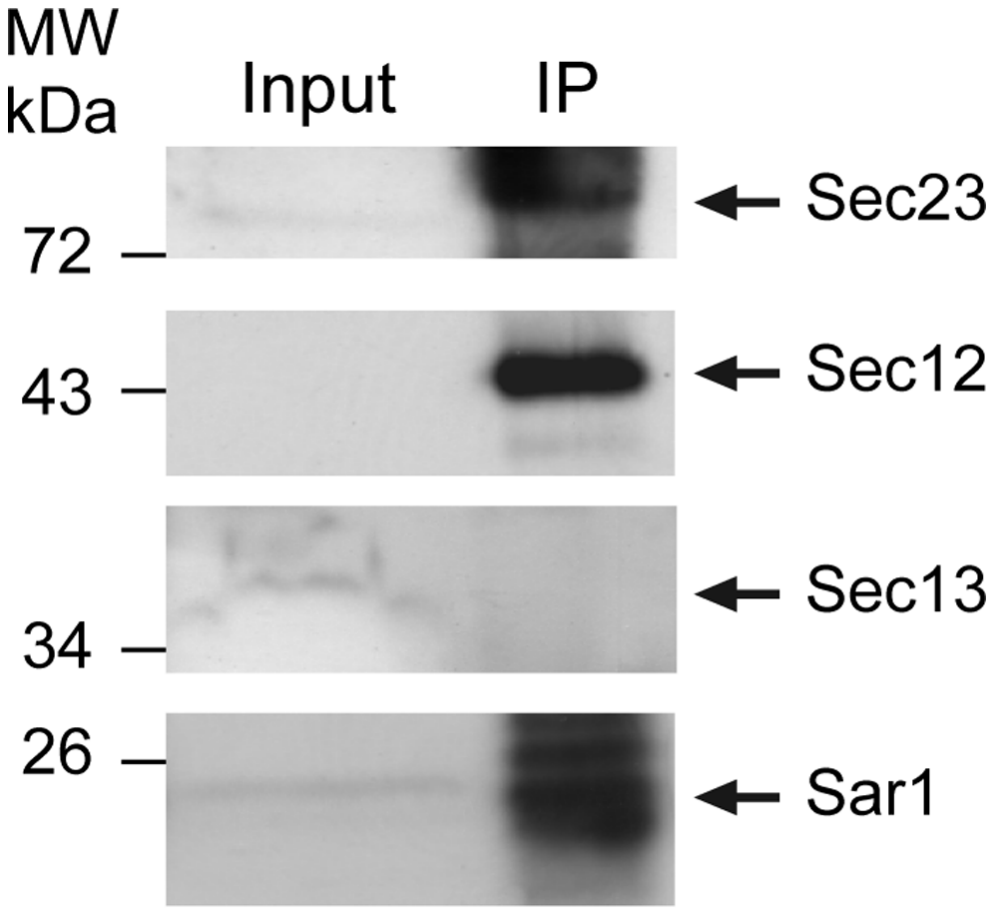
Immunopurification of a partial COPII complex in plant cells. Triton-X100 extract of 15 day-old seedlings of Col-0 *A. thaliana* was immunoprecipitated with anti-AtSar1B serum. The resulting immunoprecipitate was collected with Protein A/Protein G/γ-Bind-Sepharose beads and analyzed by Western blotting using anti-AtSar1B, anti-AtSec12, anti-AtSec13A, anti-AtSec23B serum. A total cell extract was added as control (Input).

Our yeast complementation analysis led to the identification of the following plant homologs: AtSar1D (At1g09180), AtSec12 (At2g01470, previously identified by [11]), AtSec24A (At3g07100) and AtSec13A (At3g01340) (Table 1). These results suggest that in yeast cells, these plant proteins can be associated to the other yeast COPII components to form a functional COPII coat. To determine whether *in planta* COPII proteins could also be associated in a complex, we did a co-immunoprecipitation by using the anti-AtSar1B serum (Fig. 5). Total Triton X-100 extracts of 15-day-old *A. thaliana* seedlings were incubated with anti-AtSar1B antibodies, and the precipitates were analyzed by Western blotting using the available anti-AtSec12, -AtSec13A, or -AtSec23B antibodies (Fig. 5). We found that AtSec12 and AtSec23 were co-immunoprecipitated with AtSar1, whereas AtSec13 proteins did not. This demonstrates that AtSar1, AtSec12 and AtSec23 can form a protein complex that might represent an active COPII complex in plants.

**Table 1:** Summary of the COPII complementation experiments.

Yeast protein	Plant protein	Plant gene	Growth improvement	α-factor secretion	GFP-Snc1 SNARE secretion
Sec12	**AtSec12**	***AT2G01470***	**+**	**+**	**+**
	AtSec12L	*AT5G50550*	n.d.	n.d.	n.d.
	AtSec12L1	*AT3G52190*	n.d.	n.d.	n.d.
Sar1	AtSar1A	*AT4G02080*	−	−	−
	AtSar1B	*AT1G56330*	−	−	−
	AtSar1C	*AT3G62560*	−	−	−
	**AtSar1D**	***AT1G09180***	**+**	**+**	**+**
Sec23	AtSec23A	*AT3G23660*	−	−	−
	AtSec23B	*AT4G14160*	−	−	−
	AtSec23C	*AT1G05520*	n.d.	n.d.	n.d.
	AtSec23D	*AT2G21630*	−	−	−
	AtSec23E	*AT5G43670*	−	−	−
Sec24	**AtSec24A**	***AT3G07100***	**+**	**+**	**+**
Lst1	AtLst1A	*AT3G44340*	−	−	−
	AtLst1B	*AT4G32640*	−	−	−
Sec13	**AtSec13A**	***AT3G01340***	**+/−**	**+**	**+**
	AtSec13B	*AT2G30050*	−	−	−
Sec31	AtSec31A	*AT3G63460*	−	−	−
	AtSec31B	*AT1G18830*	n.d.	n.d.	n.d.

## Discussion

In the present work, the numerous COPII constituents found in *A. thaliana* were analyzed by heterologous expression in *S. cerevisiae* and tested for their ability to complement yeast loss-of-function mutations. This first required the identification of all *A. thaliana* COPII proteins by BLAST analysis using the corresponding yeast proteins as bait. Although most proteins were correctly annotated, by using a more recent proteome database we confirmed the existence of two Sec12 paralogs (AtSec12L/At5g50550 and PHF1/AtSec12L1/At3g52190) that were not found in the previous exhaustive analysis done by Robinson and colleagues [9]. It was previously shown that the PHF1/AtSec12L1 protein does not complement the temperature-sensitivity of the yeast *sec12-1* mutant, which was not surprising since the AtSec12L1 protein lacks most of the conserved residues essential for the COPII function of Sec12 [37]. We also found that the Sec23/24 families contained some discrepancies following our multiple sequence alignment and phylogenetic analysis of representatives of the various eukaryotic taxa (7 Metazoa, 7 Fungi and 4 plants) (Fig. S1). The *A. thaliana* At4g32640 protein previously attributed to the Iss1 family [9] rather belongs to the Lst1 family (paralogs of Sec24). We confirmed that At2g27460 and At4g01810 proteins that were previously described as being very divergent from both Sec23 and Sec24 proteins [9], do not belong to the Sec23 nor the Sec24 families despite sharing high homologies with domains of Sec23 and Sec24 proteins. These two proteins were thus renamed AtSec23/Sec24L1 for At2g27460 and AtSec23/Sec24L2 for At4g01810. This suggests that AtSec23/Sec24L1 and -L2 could constitute a new COPII protein family involved in a new plant-specific COPII-like transport. Indeed, a previous study revealed that the AtSec12L1/PHF1 protein is localized at the ER and functions in early secretory trafficking, however this AtSecL1/PHF1 protein does not play the role of a classical COPII component and is rather involved in the specific trafficking of phosphate transporters [37].

Once we have assigned the *A. thaliana* COPII proteins to their correct family, we proceeded with their systematic analysis by yeast complementation assays. Since in yeast *S. cerevisiae* all COPII proteins except Lst1 are essential for viability, we chose to analyze the plant proteins in suitable thermosensitive yeast mutants as previously done by d’Enfert and colleagues [11]. Compared to gene knockouts, conditional temperature-sensitive mutants are powerful tools to study essential genes, indeed this strategy allows to observe both a phenotypic rescue at the restrictive temperature (30, 35 or 37°C) but also an amelioration at the permissive temperature (25°C) upon the expression of the plant COPII gene. Indeed in the study by Faso *et al*.(2009), the authors did not observe a complementation of the yeast *sec24Δ* mutant by the *Arabidopsis* AtSec24A construct [31]. Whereas, in our study the AtSec24A isoform complemented the temperature-sensitive defect displayed by the *sec24-11* yeast mutant and ameliorated its secretion defect. In our study, we used as complementation criteria of improved growth at different temperatures, restoration of α-factor secretion and secretion of the plasma membrane SNARE GFP-Snc1 [11], [38], [39]. Indeed, some plant isoforms might ameliorate the secretion without being able to complement the temperature-sensitivity of the yeast loss-of-function mutant. Our yeast complementation analysis in conditional temperature-sensitive mutants led to the identification of the following plant homologues: AtSar1D (At1g09180), AtSec12 (At2g01470, previously identified by [11]), AtSec24A (At3g07100) and AtSec13A (At3g01340) (Table 1). None of the Sec23, Lst1 or Sec31 plant homologs tested were able to complement the secretion defect of the corresponding *sec23-1, lst1Δ* or *sec31-1* yeast mutant. However, among the five Sec23 plant isoforms we could not test AtSec23C (At1g05520). For Sec31 the second isoform AtSec31B that is expressed almost exclusively in the male organs (pollen and stamen) was also not tested. Thus, these two non-tested isoforms could be the ones that complement the yeast loss-of-function mutant. Alternatively, the plant Sec23 and Sec31 COPII subunits have diverged so much from their fungi homologs that they no longer recognize their binding partner from other taxa. This is consistent with the regulatory role of Sec23 as the Sar1 GTPase activating protein (GAP) moiety of the Sec23/24 complex and with the primary role of Sec31 as a structural component [40]. The absence of complementation by AtLst1A and AtLst1B of the yeast *lst1Δ* mutant is probably due to our lack of understanding of the precise role of Lst1 in yeast and we can hypothesize that Lst1, AtLst1A and AtLst1B recognize very different cargos.

In the study by D’Enfert *et al.* (1992), the authors isolated AtSar1B (At1g56330) in a multicopy suppressor screen for plant cDNAs complementing the *sec12-1* temperature-sensitive phenotype [11]. Interestingly, probing the direct suppression of the *sar1-2* mutant by AtSar1 paralogs showed that AtSar1D (At1g09180) and not AtSar1B ameliorates growth and secretion of the *sar1-2* mutant; this was not due to a lack of expression of AtSar1B since both AtSar1B and AtSar1D proteins are detected in yeast cells (Fig. 2A). Two different plant Sar1 isoforms were isolated in screens with yeast affected in two different COPII encoding genes and none of the two plant isoforms allowed the yeast cells to grow at a temperature higher than 30°C, suggesting that they do not fully complement the yeast loss-of-function. These two yeast complementation analyses suggest that these two plant Sar1 isoforms are functional in COPII vesicular trafficking but might be associated in different COPII subcomplexes. Interestingly, the intracellular localization of AtSar1B-YFP was different that the one observed for AtSar1D-YFP in transfected tobacco leaves, AtSar1B being more associated to membranes than AtSar1D [41].

Our complementation assay identified the Arabidopsis AtSec24A (At3g07100) isoform as a functional Sec24 protein rescuing the yeast *sec24-11* temperature-sensitive and secretion phenotypes. The same isoform was isolated in two different screens for *A. thaliana* mutants [31], [42]. The *sec24a/ermo2* recessive mutant was isolated in a screen for *A. thaliana* mutants that had a disorganized ER morphology [42]. The other *sec24A* mutant was isolated in a screen for mutants stably expressing the Golgi specific marker ST-GFP, the *sec24A* mutant presenting a large fluorescent globular structure in addition to Golgi stacks [31]. Interestingly, these two screens identified the same mutation *sec24A^R693K^* located in a highly conserved region and that is responsible for cargo binding in *S. cerevisiae* [21], [42]. This suggests that focusing the study of either point mutant or deletion mutant of plant genes identified as being functional in our yeast complementation assay might allow to better understand the role of the COPII coat in plant cells.

Finally our identification of four *A. thaliana* COPII proteins able to complement yeast mutants suggest that they could be associated in functional COPII complex(es) in plant. Indeed, we could demonstrate that AtSar1, AtSec12 and AtSec23 are associated *in vivo* in plants. Interestingly, in our yeast analysis we could not find an AtSec23 protein that complemented the defect of the yeast *sec23-1* mutant strain, whereas AtSec23 is associated to this partial COPII complex immunopurified from plant seedlings. This suggests that the plant AtSec23 protein is evolutionary divergent from its yeast homolog and cannot functionally complement the *sec23-1* mutant. AtSec13 was not immunopurified with this partial plant COPII complex, despite its complementation of the yeast *sec13-1* mutant strain, this could be due to the experimental conditions that did not allow its immunoprecipitation. In this immunopurified plant COPII complex, we cannot ascertain which plant isoform is included into the COPII complex since we used polyclonal antibodies that recognize several isoforms (Fig. 2A, Fig. 4A and Fig. S3A). In the future, identification of the co-variations of the interaction sites between the identified functional plant COPII subunits coupled with the expression profile of the corresponding genes could predict the various complex permutations and this should allow a better understanding of the presence of so many paralogs in the plant genomes.

## Materials and Methods

### Strains, Media and Genetic Manipulations

Standard methods were used for cell growth, DNA manipulations and transformations. *E. coli* strain DH5α was used for plasmid propagation. Bacteria were grown in LB media supplemented with the appropriate antibiotic. Yeast cells were grown at 25°C in rich medium (YPD): 1% yeast extract, 2% peptone, 2% glucose or on synthetic medium (SC): 0,67% yeast nitrogen base without amino acids, 2% glucose and the appropriate dropout mix. Strains used in this study are listed in Table S1. Yeast transformants were selected on SC medium with the appropriate amino acids mixture to ensure plasmid maintenance. Yeast temperature-sensitive mutant cells were grown in the appropriate medium at 25°C, 30°C, 35°C or 37°C.

### Multiple Sequence Alignment

Amino acid sequences of proteins Sec12, Sar1, Sec23, Sec24, Sec13 and Sec31 were gathered for 7 Metazoa (*Homo sapiens, Danio rerio, Xenopus tropicalis, Drosophila melanogaster, Caenorhabditis elegans, Strongylocentrotus purpuratus* and *Ciona intestinalis*), 7 Fungi (*Saccharomyces cerevisiae, Ashbya gossypii, Schizosaccharomyces pombe, Candida glabrata, Kluyveromyces lactis, Debaryomyces hansenii, Yarrowia lipolytica*), 4 plants (*Arabidopsis thaliana, Vitis vinifera, Populus trichocarpa* and *Oryza sativa*), from the NCBI databank. Sequences were blasted using BLASTP, TBLASTN and PSI-BLAST to ascertain the absence of any missing protein [43] and aligned using PipeAlign cascade [44] and were manually adjusted.

### Phylogeny

In order to reconstruct the phylogeny, we used the full length Sar1, Sec12, Sec13, Sec23 and the most conserved region between amino acids 174 to 685 of the yeast Sec24 and 1 to 334 of the yeast Sec31. We used the neighbor-joining algorithm implemented in Seaview with 500 bootstraps to generate the phylogenetic tree [45]. For tree visualization and editing we used iTOL [46].

### Plasmid Construction

All plasmids were constructed using the Gateway (Invitrogen) cloning system. The *A*. *thaliana* genes listed in Table 1 were cloned in the donor vectors pDONRZeo or pDONR201 and recombined in the low copy number centromeric plasmid (CEN, pVV204 or pVV208, 1 to 3 copies per cell) allowing the expression of genes under the control of the tetracycline-repressible *tetO* promoter [47]. The other plasmids used are pJMG118 (GFP-Snc1 *URA3*), pJMG176 (GFP-Snc1, *LEU2*) and pJMG129 (GFP-Snc1 *TRP1*) [26]. The plasmids are described in Table S1.

### Total Protein Extracts

Equivalent of 1.5 OD of cells were harvested and lysed in 500 µl of NaOH 0.185 M for 10 minutes on ice. 50 µl of TCA 50% were added and incubated for 10 minutes on ice. Extracts were spun for 5 minutes at 12000 g. Pellets were resuspended in 50 µl of loading buffer and heated at 37°C for 10 minutes. 10 µl of protein extract were separated by PAGE and after transfer immunoblotted with the appropriate antibodies anti-AtSec12 and anti-AtSar1 [18], anti-AtSec23 [30], anti-Sec13 [35] and anti-GFP (Roche). The ratio of phosphorylated GFP-Snc1 versus total GFP-Snc1 was determined after quantification of the corresponding bands on the western-blot by using the ImageJ software (National Institutes of Health, Bethesda, MD).

### Yeast Temperature-sensitive Complementation Assay

Yeast cell cultures were grown overnight at 25°C to an OD_600 nm_ of 0.6–0.8 and harvested by centrifugation prior to be diluted to an OD_600 nm_ of 0.5. This cell suspension was serially diluted by a factor 10 until 5.10^−6^ OD_600 nm_. Then 5 µl of each cell suspension was spotted on selective media and plates were incubated at 25°C, 30°C, 35°C and 37°C for 48 hours.

### α-factor Secretion Test

Yeast cell cultures were grown overnight at 25°C to an OD_600 nm_ of 0.6–0.8 and the equivalent of 2.5 OD_600 nm_ were harvested by centrifugation and resuspended in 25 µl of medium from which 5 µl was spotted onto *bar1*-plates. These plates were made as follows, the α-factor sensitive strain RH448 (*MATa bar1*) was grown in rich medium and 800 µl of RH448 cells suspension at OD_600 nm_ 0.5 were added to 150 ml of liquid YPD-agar at 50°C before pouring plates. Spotted plates were incubated at 25°C, 30°C, 35°C and 37°C for 48 hours.

### Microscopy

Cells expressing GFP-Snc1 were grown at the permissive temperature (25°C) to mid-exponential growth phase in selective medium and shifted or not to another temperature (as indicated) before observation in the selective medium using fluorescence microscopy (Axiovert 200, 100× objective, differential interference contrast (DIC) and GFP filters [Carl Zeiss, Jena, Germany]). Images were acquired with AxioVision (Zeiss) software using a CoolSnapHQ2 camera (Roper Scientific, Tucson, AZ) and processed with ImageJ software (National Institutes of Health, Bethesda, MD).

### Immunoprecipitation on *A. thaliana* Protein Extracts

*A. thaliana* Col-0 strain was cultivated on Petri dishes with half strength of Murashige–Skoog plant medium. 15-day-old *A. thaliana* seedlings were collected and 900 mg were frozen in liquid N_2_ prior protein extraction by grinding into powder in liquid N_2_ using a mortar and pestle and further grinded after the addition of 900 µl of lysis buffer (20 mM HEPES, pH6,8, 125 mM potassium acetate, 1 mM EDTA, Triton-X100 1% and the following protease inhibitors, PMSF 1 mM and Complete™ protease inhibitor mixture tablets (Roche)) on ice. The total protein extract was first incubated 1 hr at 4°C on a slow rotating wheel then cleared by centrifugation at 10000 g for 10 min at 4°C. The supernatant was collected and pre-cleared by incubation for 30 min with 25 µl of a mix of protein A/protein G/γ-Bind-Sepharose beads (1∶1∶1 slurry) before being subjected to immunoprecipitation, 10 µl was used as the Input fraction for the western-blot analysis. Immunoprecipitation was performed over-night at 4 °C on a slow rotating wheel with 900 µl of pre-cleared Triton X-100 plant cell extracts using 5 µl of anti-AtSar1 serum and 50 µl of a mix of protein A/protein G/γ-Bind-Sepharose beads (1∶1∶1 slurry) in a total volume of 1 ml. The immunoprecipitate was collected by centrifugation, washed five times with lysis buffer and once in 20 mM Tris, pH 6.8. This co-immunoprecipitation protocol was based on the protocol described by Pagano et al. (1999) used to determine the interactions between hSec23 and hSec24 proteins in 293T cells [48]. The beads were resuspended in SDS sample buffer and then run on 10% polyacrylamide gels before being transferred to nitrocellulose. The western-blot was revealed with anti-AtSar1 (d = 1/500), anti-AtSec13 (d = 1/70 of immunopurified serum), anti-AtSec12 (d = 1/500) and anti-AtSec23 (d = 1/2000) serum, followed by incubation with secondary HRP-coupled antibodies and ECL detection.

## Supporting Information

Figure S1 **Phylogeny of Sec12, Sar1, Sec23, Sec24, Sec13 and Sec31 proteins from the Metazoa, Fungi and Plantae phyla.** For each family, protein sequences from 6 representatives of each phylum were aligned and the most conserved portion was used to calculate the phylogenetic tree using SeaView by parsimony with 500 bootstrapped replications. The tree display was performed by iTOL and the tree re-rooted at the base of the Plantae branch. (*) labels the isoform suppressing the corresponding yeast mutation.
(TIF)

Figure S2 **Complementation by plant COPII encoding genes of the temperature-sensitive phenotype of yeast COPII mutants.** The indicated *S. cerevisiae* COPII thermosensitive mutant strains were transformed with either the empty vector (pVV) or with the pVV plasmid bearing the indicated *A. thaliana* isoform. A drop (5 µl) of ten-fold serial dilutions of 0.5 OD_600 nm_ of the different yeast cells cultures grown to mid-exponential phase at 25°C were spotted on YPD medium to determine the growth at permissive (25°C) and various restrictive temperatures (30°C, 35°C and 37°C) after a 2–3 days incubation at the indicated temperature.
(TIF)

Figure S3 **Analysis of the yeast** ***sec23-1*** **mutant complementation by the plant** ***AtSEC23/24L*** **and At** ***SEC23*** **A, B, D and E isoforms.** A) Total protein extracts of *A. thaliana* leafs (Ara), *S. cerevisiae sec23-1* mutant cells (*sec23*) untransformed or transformed with empty vector (pVV) or the *Arabidopsis* At*SEC23* A, B, D and E isoforms as well as *AtSEC23/24L* were resolved by SDS-PAGE and immunoblotted with anti-AtSec23B antibodies. B) Cell cultures grown at 25°C to mid-exponential phase of the same strains as in A) were spotted on YPD medium containing *MATa bar1* mutant cells to determine α-factor secretion at permissive (25°C) and various restrictive temperatures (30°C and 35°C). C) The intracellular localization of the COPII cargo GFP-Snc1 was determined by fluorescence microscopy in the *sec23-1* mutant cells transformed with either pVV or A, B, D and E isoforms of AtSec23 as well as AtSec23/24L plant isoforms and grown to mid-exponential phase at 25°C. D) Total proteins were extracted from the same cultures as the ones described in C) and the phosphorylation status of GFP-Snc1 was detected by anti-GFP western-blot.
(TIF)

Figure S4 **Analysis of the complementation of the yeast** ***lst1Δ*** **strain by the plant At** ***SEC24*** **A,** ***AtLST1A*** **and** ***-B*** **as well as** ***AtSEC23/24L*** **isoforms.** A) *lst1Δ* mutant cells were transformed with either the empty vector (pVV) or pVV plasmids bearing the plant At*SEC24*A, *AtLST1A* and *-B* as well as *AtSEC23/24L*. A 5 µl drop of ten-fold serial dilutions of 0.5 OD_600nm_ cell suspensions was spotted on YPD plates and incubated at 25°C, 30°C, 35°C and 37°C for 2–3 days. B) Cells of the same strains were spotted on YPD medium containing *MATa bar1* mutant cells to determine α-factor secretion at 25°C, 30°C, 35°C and 37°C. C) The localization of the COPII cargo GFP-Snc1 was determined in the same strains after co-transformation with the GFP-Snc1 vector and growth to mid-exponential phase at 25°C. D) Total protein extracts of the same strains as in C) were resolved by SDS-PAGE and immunoblotted with anti-GFP antibodies.
(TIF)

Figure S5 **Analysis of complementation of the yeast** ***sec31-1*** **mutant by the plant At** ***SEC31A*** **.** A) Wild-type (WT) and *sec31-1* mutant cells untransformed or transformed with empty vector (pVV) or At*SEC31A* were spotted on YPD medium containing *MATa bar1* mutant cells to determine α-factor secretion at permissive (25°C) and various restrictive temperatures (30°C, 35°C and 37°C). B) The *sec31-1* mutant cells transformed with pVV or At*SEC31A* plasmids and with GFP-Snc1 vector were grown at 25°C to mid-exponential phase, then half of the cultures was incubated at 25°C or 37°C for 2 hr, before being analyzed by fluorescence microscopy to observe the localization of GFP-Snc1. C) The same cultures treated as described in B) were lysed and the total protein extract was resolved by SDS-PAGE and immunoblotted with anti-GFP antibodies.
(TIF)

Table S1 **Yeast strains and plasmids.**
(DOC)
