# Supplementary figures and images for: Study of the Plant COPII Vesicle Coat Subunits by Functional Complementation of Yeast Saccharomyces cerevisiae Mutants
Source: PLoS One. 2014 Feb 25;9(2):e90072. doi: 10.1371/journal.pone.0090072 (PMC3934973; doi:10.1371/journal.pone.0090072)

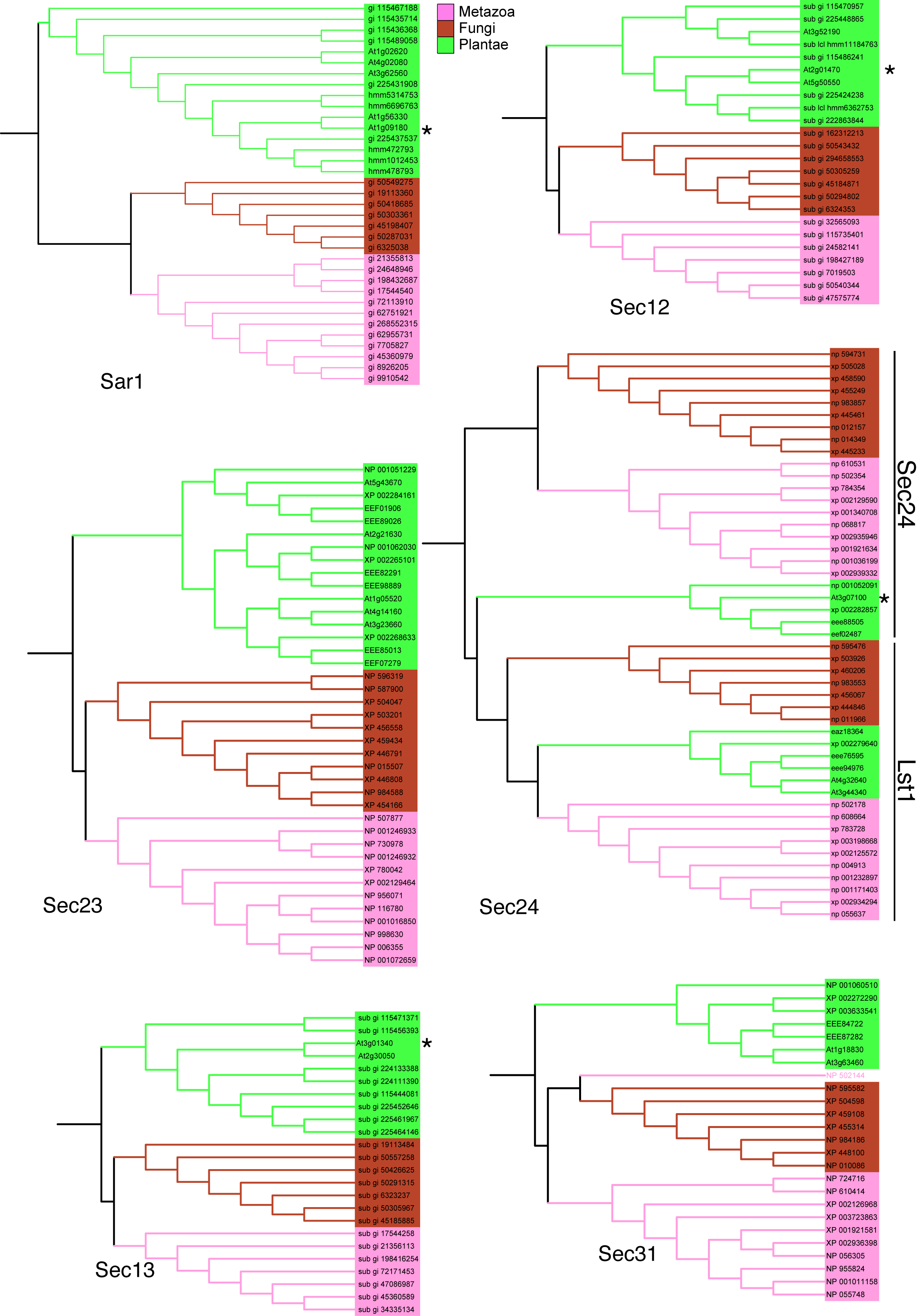

Supplement: Figure S1 — Phylogeny of Sec12, Sar1, Sec23, Sec24, Sec13 and Sec31 proteins from the Metazoa, Fungi and Plantae phyla. For each family, protein sequences from 6 representatives of each phylum were aligned and the most conserved portion was used to calculate the phylogenetic tree using SeaView by parsimony with 500 bootstrapped replications. The tree display was performed by iTOL and the tree re-rooted at the base of the Plantae branch. (*) labels the isoform suppressing the corresponding yeast mutation. (TIF) [file pone.0090072.s001.tif]

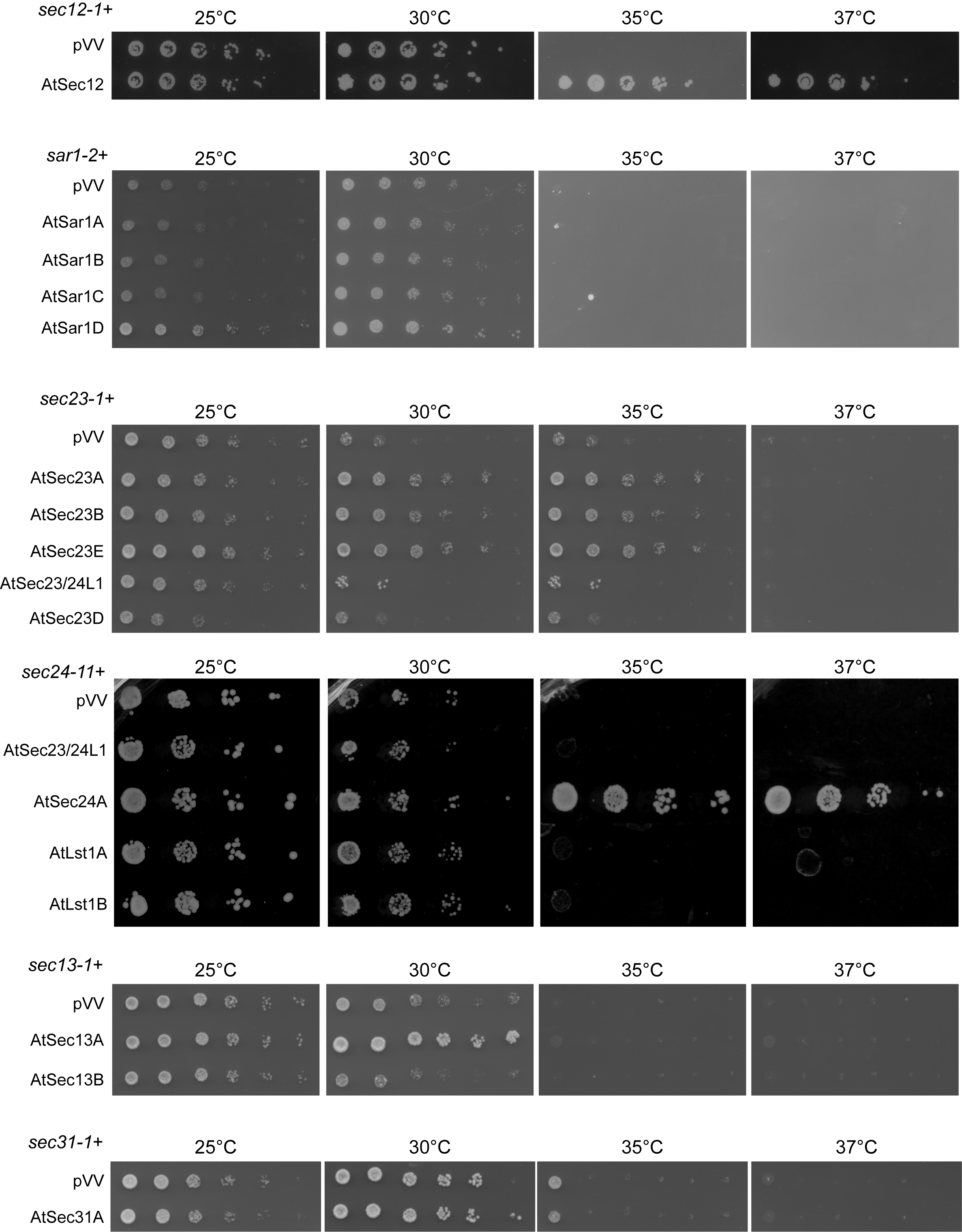

Supplement: Figure S2 — Complementation by plant COPII encoding genes of the temperature-sensitive phenotype of yeast COPII mutants. The indicated S. cerevisiae COPII thermosensitive mutant strains were transformed with either the empty vector (pVV) or with the pVV plasmid bearing the indicated A. thaliana isoform. A drop (5 µl) of ten-fold serial dilutions of 0.5 OD600 nm of the different yeast cells cultures grown to mid-exponential phase at 25°C were spotted on YPD medium to determine the growth at permissive (25°C) and various restrictive temperatures (30°C, 35°C and 37°C) after a 2–3 days incubation at the indicated temperature. (TIF) [file pone.0090072.s002.tif]

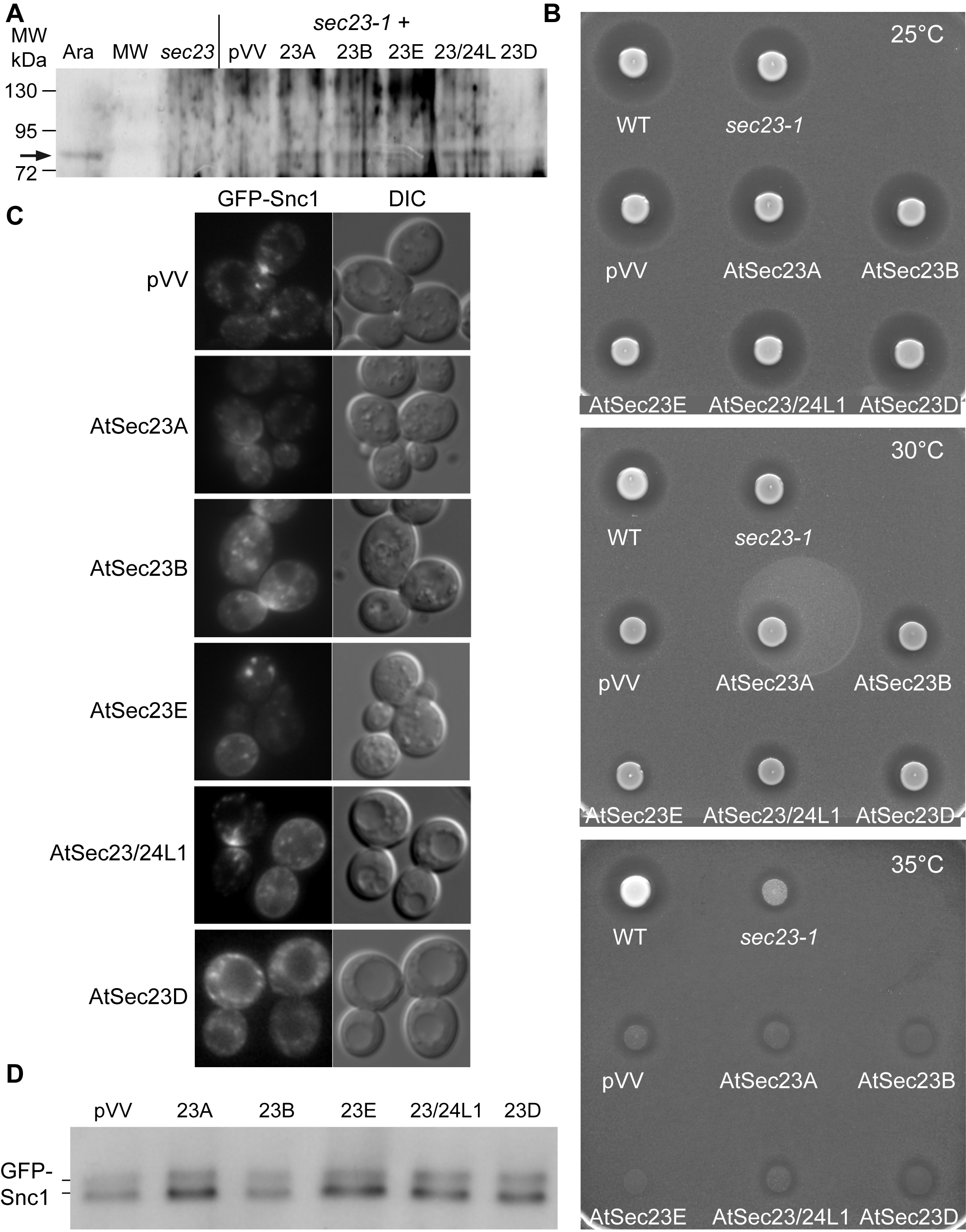

Supplement: Figure S3 — Analysis of the yeast sec23-1 mutant complementation by the plant AtSEC23/24L and At SEC23 A, B, D and E isoforms. A) Total protein extracts of A. thaliana leafs (Ara), S. cerevisiae sec23-1 mutant cells (sec23) untransformed or transformed with empty vector (pVV) or the Arabidopsis AtSEC23 A, B, D and E isoforms as well as AtSEC23/24L were resolved by SDS-PAGE and immunoblotted with anti-AtSec23B antibodies. B) Cell cultures grown at 25°C to mid-exponential phase of the same strains as in A) were spotted on YPD medium containing MATa bar1 mutant cells to determine α-factor secretion at permissive (25°C) and various restrictive temperatures (30°C and 35°C). C) The intracellular localization of the COPII cargo GFP-Snc1 was determined by fluorescence microscopy in the sec23-1 mutant cells transformed with either pVV or A, B, D and E isoforms of AtSec23 as well as AtSec23/24L plant isoforms and grown to mid-exponential phase at 25°C. D) Total proteins were extracted from the same cultures as the ones described in C) and the phosphorylation status of GFP-Snc1 was detected by anti-GFP western-blot. (TIF) [file pone.0090072.s003.tif]

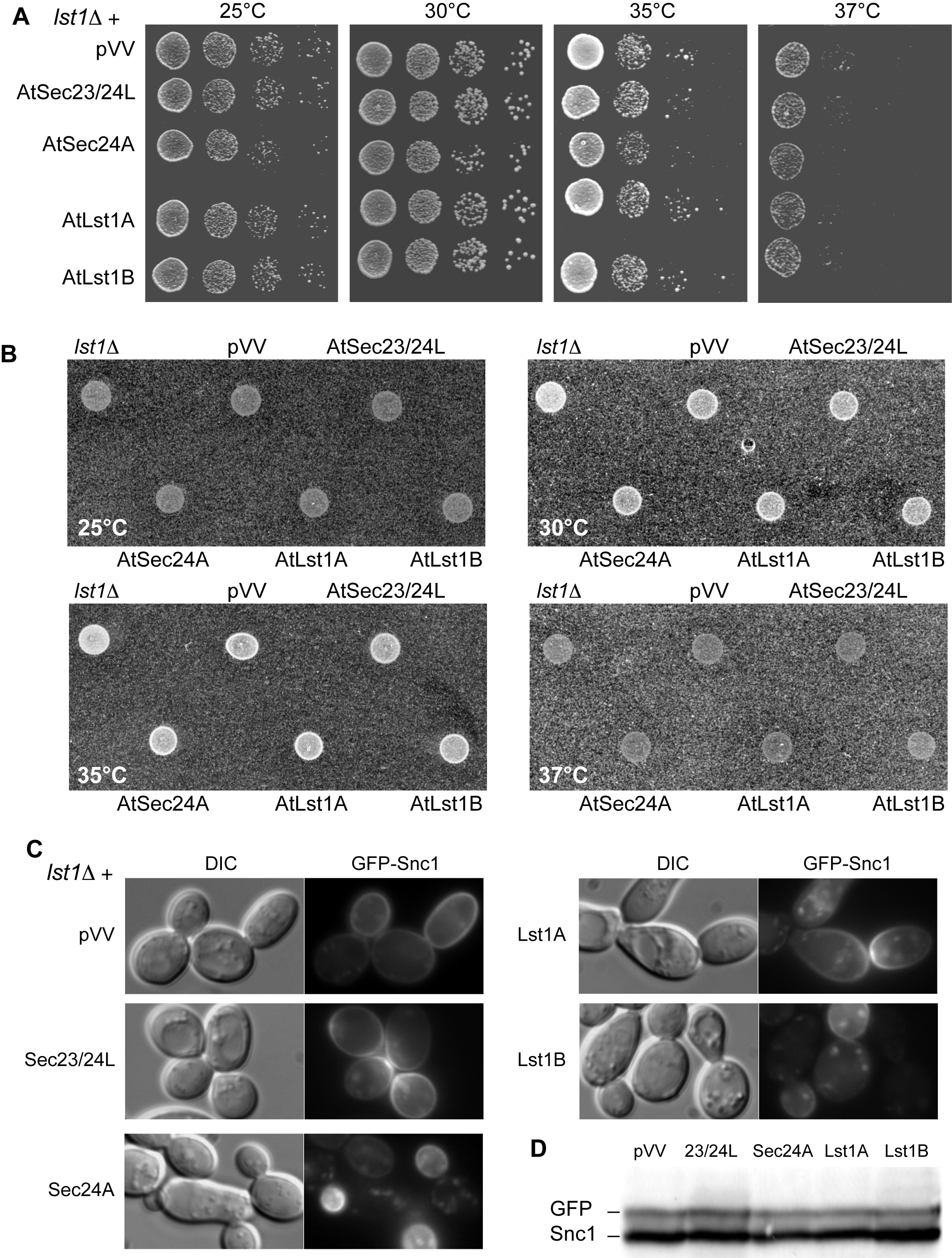

Supplement: Figure S4 — Analysis of the complementation of the yeast lst1Δ strain by the plant At SEC24 A, AtLST1A and -B as well as AtSEC23/24L isoforms. A) lst1Δ mutant cells were transformed with either the empty vector (pVV) or pVV plasmids bearing the plant AtSEC24A, AtLST1A and -B as well as AtSEC23/24L. A 5 µl drop of ten-fold serial dilutions of 0.5 OD600nm cell suspensions was spotted on YPD plates and incubated at 25°C, 30°C, 35°C and 37°C for 2–3 days. B) Cells of the same strains were spotted on YPD medium containing MATa bar1 mutant cells to determine α-factor secretion at 25°C, 30°C, 35°C and 37°C. C) The localization of the COPII cargo GFP-Snc1 was determined in the same strains after co-transformation with the GFP-Snc1 vector and growth to mid-exponential phase at 25°C. D) Total protein extracts of the same strains as in C) were resolved by SDS-PAGE and immunoblotted with anti-GFP antibodies. (TIF) [file pone.0090072.s004.tif]

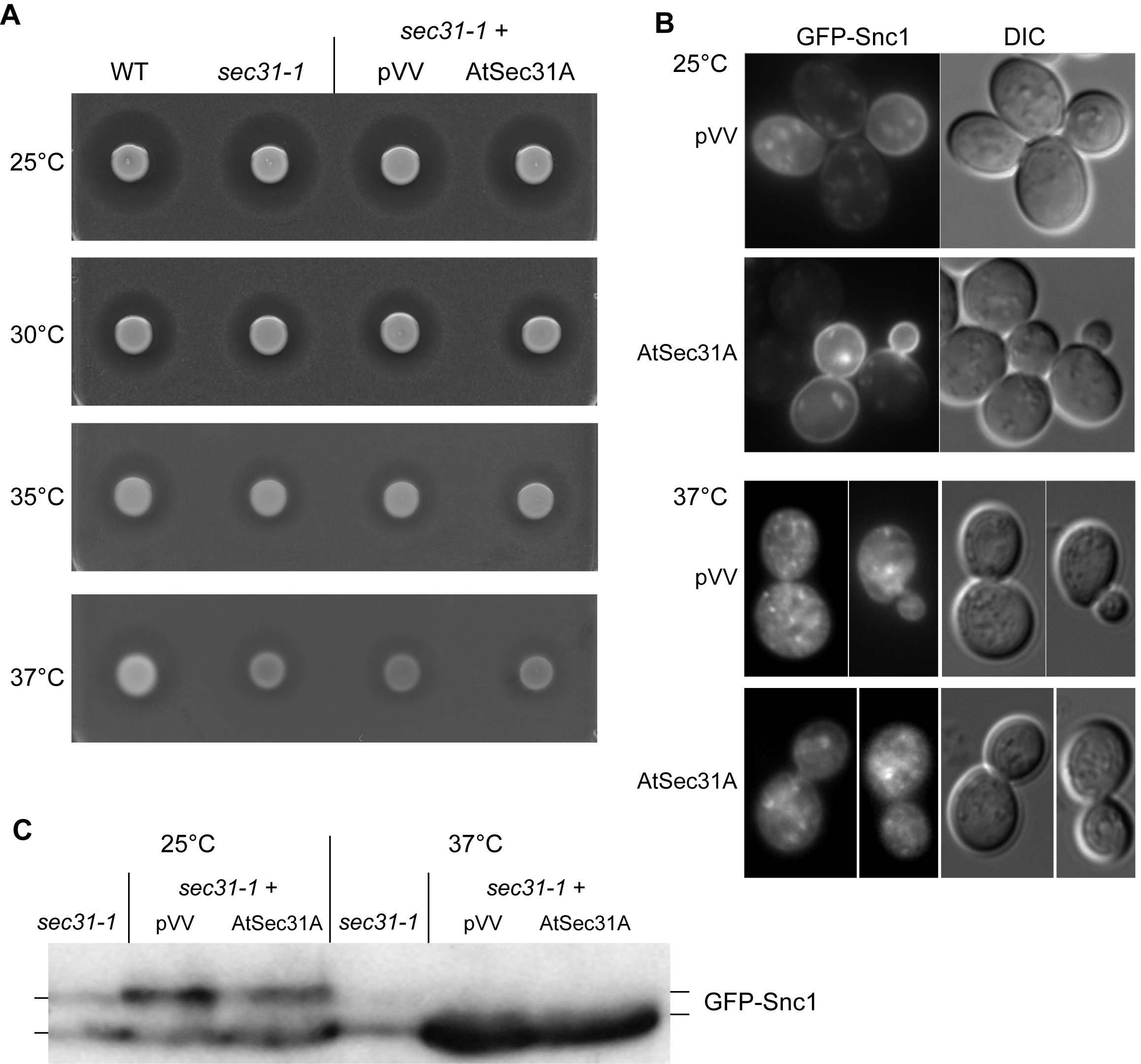

Supplement: Figure S5 — Analysis of complementation of the yeast sec31-1 mutant by the plant At SEC31A . A) Wild-type (WT) and sec31-1 mutant cells untransformed or transformed with empty vector (pVV) or AtSEC31A were spotted on YPD medium containing MATa bar1 mutant cells to determine α-factor secretion at permissive (25°C) and various restrictive temperatures (30°C, 35°C and 37°C). B) The sec31-1 mutant cells transformed with pVV or AtSEC31A plasmids and with GFP-Snc1 vector were grown at 25°C to mid-exponential phase, then half of the cultures was incubated at 25°C or 37°C for 2 hr, before being analyzed by fluorescence microscopy to observe the localization of GFP-Snc1. C) The same cultures treated as described in B) were lysed and the total protein extract was resolved by SDS-PAGE and immunoblotted with anti-GFP antibodies. (TIF) [file pone.0090072.s005.tif]
