# Supplementary material for: Study of the Plant COPII Vesicle Coat Subunits by Functional Complementation of Yeast Saccharomyces cerevisiae Mutants
Source: PLoS One. 2014 Feb 25;9(2):e90072. doi: 10.1371/journal.pone.0090072 (PMC3934973; doi:10.1371/journal.pone.0090072)
Supplement: Table S1 — Yeast strains and plasmids. (DOC) [file pone.0090072.s006.doc]

| **Strains** | **Genotype** | **Reference** |
| --- | --- | --- |
| SEY6210 | MAT**α** *his3 leu2 lys2 ura3 trp1 suc2* | Scott D. Emr |
| RH448 | MAT**a** *his4 leu2 ura3 lys2 bar1* | Howard Riezman |
| RH449 | MAT**α** *his4 leu2 ura3 lys2 bar1* | Howard Riezman |
| *sar1-2* | MAT**α** *ura3 leu2 trp1 his3 ade2 sar1::HIS3 pep4::ADE2* [YCp-*sar1-2 LEU2*] | [28] |
| *sec12-1* | MAT**α** *ura3 his4 leu2 lys2 sec12-1* | [1] |
| *sec13-1* | MAT**α** *ura3 his4 sec13-1* | [1] |
| *sec23-1* | MAT**α** *ura3 leu2 sec23-1* | [1,29] |
| *sec24-11* | MAT**α** *ura3 leu2 his3 trp1 ade2 sec24-11* | [32] |
| *sec31-1* | MAT**α** *ura3 leu2 sec31-1* | [36] |
| *lst1*Δ | MAT**a** *his3 leu2 lys2 ade2 ura3 trp1 lst1::HIS3* | [33] |
| SFY89 | MAT**α** *ura3 trp1 lys2 his3 his4 sec13-1* | This study |
| SFY144 | MAT**α** *ura3 trp1 lys2 leu2 his3 lst1::HIS3* | This study |
| **Plasmids** | **Description** | **Reference** |
| pDONR201 | Entry vector Gateway®, Kan^R^ | Invitrogen |
| pDONR-Zeo | Entry vector Gateway®, Kan^R^ | Invitrogen |
| pVV204 | Destination yeast expression vector Gateway®, CEN, *TRP1*, TetO prom | [47] |
| pVV208 | Destination yeast expression vector Gateway®, CEN, *URA3*, TetO prom | [47] |
| pJMG118 | GFP-Snc1, *URA3* | [26] |
| pJMG129 | GFP-Snc1, *TRP1* | [26] |
| pJMG176 | GFP-Snc1, *LEU2* | [26] |

Table S1: Yeast strains and plasmids
